# The effect of structured checklist-assisted multimedia interactive education on postoperative pain management and quality of life in patients with lower extremity varicose veins: a randomized controlled trial with 1-year follow-up

**DOI:** 10.3389/fpubh.2026.1753287

**Published:** 2026-03-12

**Authors:** Jing Huang, Ling Li, Min Li, Li Ren, Yukui Ma, Huanrui Hu

**Affiliations:** 1 Division of Vascular Surgery, Department of General Surgery, West China Hospital, Sichuan University, Chengdu, China; 2 West China School of Nursing, Sichuan University, Chengdu, China

**Keywords:** checklist, lower extremity varicose veins, multimedia, pain management, patient education, quality of life

## Abstract

**Objective:**

Lower extremity varicose veins (LEVV) are common chronic venous disorders. Adherence to perioperative self-care plays a vital role in managing postoperative pain and enhancing long-term quality of life (QoL). Traditional health education models often struggle with issues such as incomplete information and high cognitive load.

**Methods:**

This study utilized a single-blind, randomized controlled trial design. LEVV patients were randomly assigned to either an intervention group [checklist-enhanced multimedia interactive education (CE-MIE), *n* = 96] or a control group [multimedia interactive education (MIE), *n* = 97]. Both groups received standard perioperative care and multimedia educational resources. The intervention group also used a structured checklist for a comprehensive, bidirectional verification of educational content (including ankle pump exercises, limb elevation and discharge instructions) and key skills, with patients required to score >80 points on elastic stocking wearing skills. Primary outcomes included QoL scores, pain levels, and complication rates.

**Results:**

The CE-MIE group showed significantly better QoL scores at 1 month postoperatively compared to the MIE group (32.74 ± 4.72 vs. 35.49 ± 4.01, *p* < 0.001). Additionally, the CE-MIE group reported lower pain scores on the 3rd and 7th postoperative days. However, there were no significant differences in QoL scores between the two groups at the 1-year follow-up, and long-term pain assessment at 1 year was not included in the study design as the primary focus was on acute recovery.

**Conclusion:**

CE-MIE is an effective short-term intervention for improving QoL and pain management in LEVV patients. To address the challenge of long-term decay in intervention effectiveness, future studies should explore ways to extend the “in-hospital standardization” model to promote “out-of-hospital sustainability.”

## 1 Introduction

The rising global prevalence of chronic venous disease (CVD), driven by an aging population, lifestyle changes, and increasing obesity rates, poses a significant burden on both patients and healthcare systems (1). Epidemiological surveys conducted across 23 countries have reported varying prevalence rates for CVD: 52% in Asia, 62% in Western Europe, 68% in Latin America, and 70% in Eastern Europe (2). Lower extremity varicose veins (LEVV), a common manifestation of CVD, affect approximately 21.8–29.4% of adults and have become a major public health concern worldwide (3, 4). Patients with LEVV often experience symptoms ranging from pain and fatigue to itching and venous ulcers, all of which severely impact their quality of life (QoL) (5–8).

Patients with (LEVV primarily experience venous-related pain, characterized by a dull ache and heaviness due to venous hypertension and blood pooling (9). This chronic discomfort often worsens during the perioperative period, as surgical trauma-induced inflammatory pain overlaps with pre-existing venous symptoms (10). Additionally, pain remains one of the main postoperative symptoms (11, 12).

Pain is highly prevalent during the perioperative period and is closely associated with a patient’s overall QoL (13). QoL reflects subjective health status and disease burden, making it a key patient-reported outcome and an important indicator of clinical efficacy and long-term prognosis (14). While surgery or endovenous treatment remains the primary approach for LEVV, effective perioperative management and diligent self-care after discharge are crucial in preventing complications and reducing the risk of recurrence (15).

The postoperative rehabilitation process for LEVV patients requires mastery of several essential self-care skills, including proper limb elevation, ankle pump exercises, and the use of medical compression stockings. Poor adherence to these practices or insufficient mastery can lead to severe complications such as deep vein thrombosis (DVT) and compromise long-term treatment outcomes (16). However, systematic reviews indicate that non-adherence rates remain high due to physical limitations, discomfort, financial issues, psychosocial issues, and a lack of patient “health literacy” regarding the rationale behind these recommendations (17).

Furthermore, traditional education fails to address the “knowledge-action gap,” where patients understand the instructions but lack the self-efficacy to execute them correctly. Traditional health education models, which primarily rely on verbal instruction and paper-based handouts, have several limitations. These models often lack standardization, making the quality of care dependent on the nurse’s individual experience and time constraints, resulting in gaps in the knowledge and skill training provided to patients (18, 19). Furthermore, traditional methods do not incorporate a quantitative assessment of skill proficiency, making it challenging to ensure that patients apply their knowledge correctly. As a result, there is a pressing need for a standardized, proactive, and repeatable educational model.

To overcome the limitations of traditional models, Multimedia Education presents a promising solution due to its repeatability and high consistency (20). The structured visual and auditory content in multimedia interactive education (MIE) enhances patient learning by reducing the cognitive load associated with complex instructions, a key principle of Cognitive Load Theory (21). However, most existing multimedia education initiatives primarily focus on the one-way transfer of knowledge (22). There is a notable absence of standardized mechanisms to objectively assess whether patients have effectively translated this information into the complex skills necessary for effective self-care. For example, while a patient may comprehend the importance of wearing medical compression stockings as demonstrated in a video, they may still lack the technical skills necessary for proper application. To bridge this gap, a transition is needed from passive information delivery to an active verification model. This model should ensure that each step of skill acquisition is mutually confirmed and incorporate competency-based assessments, where a quantitative proficiency threshold must be achieved prior to patient discharge (23).

This study aimed to develop a checklist-enhanced multimedia interactive education (CE-MIE) model, which incorporates a structured checklist for full-process, two-way verification and quantitative compliance requirements for patient education and self-care skills. The CE-MIE model was designed to address the issues of information omission, poor comprehension, and lack of self-efficacy in discharge education. The objective of this study was to evaluate the effectiveness of the CE-MIE model in improving postoperative pain management and QoL compared to the standard MIE model.

## 2 Methods

### 2.1 Procedure

This was a single-blind, randomized, parallel-group trial, approved by the Ethics Committee of West China Hospital (Approval No. 2021-273; Date of Approval: March 24, 2021). The study was conducted at the same institution. Patients were selected based on the diagnosis of Great Saphenous Vein (GSV) insufficiency, categorized according to the Clinical, Etiological, Anatomical, and Pathophysiological (CEAP) classification system, with clinical classes ranging from C2 to C5. All patients underwent Radiofrequency Ablation (RFA) surgery by an experienced physician, following a standardized protocol (ClosureFast; Medtronic, Minneapolis, MN) (24). Written informed consent was obtained from all participants.

The trial was conducted, and all participants were recruited between October 2021 and February 2024. Each patient underwent venous duplex ultrasound to confirm GSV insufficiency. Key exclusion criteria were: 1) Recurrent varicose veins, deep vein thrombosis, or CEAP class C6 disease; 2) Presence of severe systemic diseases, such as kidney, liver, or cardiovascular conditions.

Patients were randomly assigned to either the intervention or the control group using a computer-generated random sequence. Research personnel, who were not involved in the intervention, managed the randomization process, ensuring that both participants and the responsible nurses remained blinded to group allocation until health education began.

### 2.2 Health education protocols

#### 2.2.1 Shared intervention content (standard care and resources)

Both groups had access to the same online health education resource platform and received basic nursing instructions.

The platform offered multimedia-based educational materials for patients and their families, accessible during hospitalization and after discharge. Text-based materials included information on the disease, pain management, the treatment process (perioperative preparation and postoperative recovery), and postoperative rehabilitation (e.g., lifestyle advice, compression stocking care). Video demonstrations covered correct execution of ankle pump exercises, proper limb elevation, and correct methods for wearing and removing compression stockings.

An open text-based question channel was available on the platform, where patients could submit queries. Research staff collected these questions nightly and provided written responses, which were posted the following morning in the “Frequently Asked Questions” (FAQ) or “Questions and Answers” (Q&A) section. Patients were advised that this Q&A mechanism was not intended for emergency medical consultation; those experiencing urgent symptoms were instructed to contact ward personnel immediately.

#### 2.2.2 Control group intervention (multimedia interactive education, MIE)

Patients in the control group received basic bedside instruction along with the shared intervention content. Nurses provided verbal education based on routine department procedures and guided patients to use the online educational resources and engage in the interactive Q&A. In this group, the multimedia platform served as a supplementary educational resource without a standardized tracking mechanism. However, nurses did not use or complete any structured checklist to track patient progress or skill mastery. Education primarily occurred at admission and discharge.

#### 2.2.3 Intervention group intervention (checklist enhanced multimedia interactive education, CE-MIE)

In addition to the shared intervention content, the intervention group utilized a structured checklist (Table 1) for comprehensive education management and skill assessment. Crucially, while both groups had identical access to the same multimedia content, the CE-MIE group was distinguished by the use of the checklist as the primary tool for process control and verification. This study was specifically designed to evaluate the efficacy of this structured checklist-assisted management model rather than the multimedia educational materials themselves.

**Table 1:** Health education checklist for varicose veins of lower extremity.

Patient name:_______ Admission date: ________ Surgery date:________ Discharge date: _______			
Item	Content	Performer/time	Patient’ signature
Admission	Ward environment: Diet, hot water, doctor’s rounds, check-ups, companion bed		
	Elastic stockings: Leg circumference measurement, confirmed purchase		
Pre-op	Confirm purchase of elastic stockings		
	Elastic stocking wearing method		
	Master ankle pump exercises, master breathing function exercises		
	Pre-operative education: Diet, medication, skin, patient gown		
	Confirm mastery of elastic stocking wearing method		
Surgical day	After returning to the ward, for those with clear consciousness, may drink about 50 mL of water; if no discomfort, may eat after 2 h (easily digestible, light, small and frequent meals)		
	Bed rest activity: Ankle pump exercises once every hour, 10 repetitions each time		
	Continuous elevation of 20–30° (place a soft pillow under the calf to avoid discomfort)		
	Pain management		
Post-op Day 2	Early ambulation: 10 min each time (no extra activity except for going to the toilet)		
	Bed rest, continuous elevation, in-bed functional exercises		
	Resume regular diet, fluid intake of over 2,000 mL		
Post-op Day 3	Ambulation, resume regular diet, fluid intake of over 2,000 mL		
	Bed rest, continuous elevation, in-bed functional exercises		
Discharge	Check elastic stocking wearing compliance		
	Check mastery of elastic stocking maintenance knowledge		
	Medication and follow-up		

All activities should be stopped immediately if discomfort is felt.

This checklist, a detailed educational and skill verification tool, was used throughout the patient’s hospitalization. It was developed by a multidisciplinary team of vascular surgeons, senior nurses, and rehabilitation therapists at West China Hospital. The development process was guided by evidence-based practices and expert consensus (25). It aimed to standardize the educational process and promote two-way interaction between nurses and patients. The checklist included items related to disease knowledge, treatment procedures, pain management, postoperative rehabilitation (ankle pump exercises, limb elevation, compression stocking wear), and discharge instructions. Nurses were required to follow the checklist strictly and complete two-way verification for each item. After a nurse completed a checklist item, both the “Nurse Confirmation” and “Patient Confirmation” columns were checked and signed. Only after all critical items were confirmed could the next stage proceed. The completed checklist was collected by the research team upon patient discharge as evidence of intervention adherence.

#### 2.2.4 Quantitative compliance for compression stocking wear

Nurses were responsible for instructing patients on the use of compression stockings and using the Compression Stocking Wear Skill Assessment Sheet (Table 2) to evaluate compliance. All patients in the intervention group were assessed preoperatively and required to score at least 80 points. Patients who did not meet this threshold received additional education and training until they achieved compliance.

**Table 2:** The Compression stocking wear skill assessment sheet.

Technical operation requirements	Scores
Nail length is appropriate.	0–5 Inappropriate—appropriate
No wristwatch or hand jewelry worn.	0–5 Worn—not worn
Worn after elevating for at least 5 min.	0–10 Unaware—partially aware—aware
Pre-wearing assessment: skin temperature, swelling, skin color; and if there are skin breaks, swelling, or redness, discontinue wearing and seek medical attention.	0–10 Unaware—partially aware—aware
The surface should remain smooth after wearing.	0–10 Unaware—partially aware—aware
One hand reaches inside the GCS sock to hold the heel area, while the other hand flips the GCS sock to the middle position of the heel and unfolds it.	0–10 Incorrect operation—partially correct—correct
Thumbs of both hands press against the inside of the sock, with the remaining four fingers gripping the outside of the GCS, inserting the foot into the sock, and coordinating both hands to pull the compression sock toward the ankle.	0–10 Incorrect operation—partially correct—correct
Align the heel of the GCS with the heel of the lower limb.	0–10 Incorrect operation—partially correct—correct
Slowly roll the sock leg back and pull it upwards.	0–10 Incorrect operation—partially correct—correct
After putting on the GCS, smooth the sock close to the skin.	0-10 Incorrect operation—partially correct—correct
With coordinated fingers of both hands, grip the inside and outside of the GCS, turn the GCS inside out, and remove it along the leg.	0–10 Incorrect operation—partially correct—correct
Wash with a neutral detergent, gently rubbing;	0–5 Unaware—partially aware—aware
Lay flat to dry, avoiding direct sunlight.	0–5 Unaware—partially aware—aware
Wear upon waking, remove before sleeping, and maintain wear duration > 12 h.	0–5 Unaware—partially aware—aware

A previous study reported that the quality of life score for patients 1 year after Radiofrequency Ablation (RFA) was 30.67 ± 8.41 (26). This study hypothesized that the new health education model would reduce the quality of life score to 27. While a specific minimal clinically important difference (MCID) for the chronic venous insufficiency questionnaire (CIVIQ) has not been established in this population, a commonly used proxy is 0.5 times the standard deviation, which equals 4.2 points in this context. To ensure a more rigorous study design, we adopted a more conservative difference of 3.67 points for our calculations. To detect this difference with 80% statistical power (*α* = 0.05, two-sided test), the required sample size per group was calculated using the formula: N1 = N2 = 2[(Zα + Zβ)*σ/*δ*]^2^, and the estimated required sample size was 83 participants per group. Accounting for a 15% dropout rate, the total sample size required was 96 participants per group.

### 2.3 Observation indexes

The primary endpoint was the assessment of quality of life using the CIVIQ at three time points: baseline, 1 month post-surgery, and 1 year post-surgery.

Secondary endpoints included:

1. The patient’s pain score, measured using the Numeric Rating Scale (NRS), at four time points: baseline, 1 day post-surgery, 3 days post-surgery, and 7 days post-surgery.
2. The incidence of local complications (ecchymosis, redness, and induration in the thigh area) at three time points: 3 days post-surgery, 7 days post-surgery, and 1 month post-surgery. Local complications were considered present if they affected more than 25% of the treated area, as defined by a standardized scale developed by Almeida et al. (27).

Quality of life was assessed using the CIVIQ, which includes 20 items across four domains: Pain, Physical, Social, and Psychological (28). The Pain domain assesses ankle or lower limb pain, interference with daily activities, impact on sleep, and limitations due to prolonged standing. The Physical domain assesses difficulties with activities such as climbing stairs, squatting, walking, and housework. The Social domain evaluates the impact on activities like riding in a small car or bus, participating in recreation, and exercising. The Psychological domain covers feelings of tension, fatigue, irritability, being a burden to others, embarrassment about showing the affected limb, difficulty getting up in the morning, impact of prolonged standing or leg extension, limping, and reluctance to go out. Each item is scored on a 1–5 scale, with a total score ranging from 20 to 100, where a higher score indicates poorer quality of life.

Pain was measured using the NRS, which ranges from 0 to 10, where 0 represents no pain and 10 represents the most severe pain. Only pain in the thigh area treated with RFA was recorded.

### 2.4 Statistical analysis

Data were analyzed using IBM SPSS Statistics version 24.0 (IBM Corp., Armonk, NY) and GraphPad Prism version 10.0.0 for Windows (GraphPad Software, Boston, MA). Figures and charts were created using GraphPad Prism. Missing data at the 1-year follow-up were handled based on the Intention-to-Treat (ITT) principle, with multiple imputation used to account for missing observations. Continuous measurement data were first tested for normality. Data following a normal distribution were summarized as mean ± standard deviation (SD) and analyzed using the independent samples *t*-test. For non-normally distributed data, results were presented as median and interquartile range (IQR) and compared using the Mann–Whitney *U* test. To control for the increased risk of Type I error due to multiple comparisons across different time points, the Bonferroni correction was applied. Categorical variables were summarized using frequency counts and percentages, with differences between groups assessed using the Chi-squared test.

## 3 Results

### 3.1 Baseline characteristics

**Figure 1:**
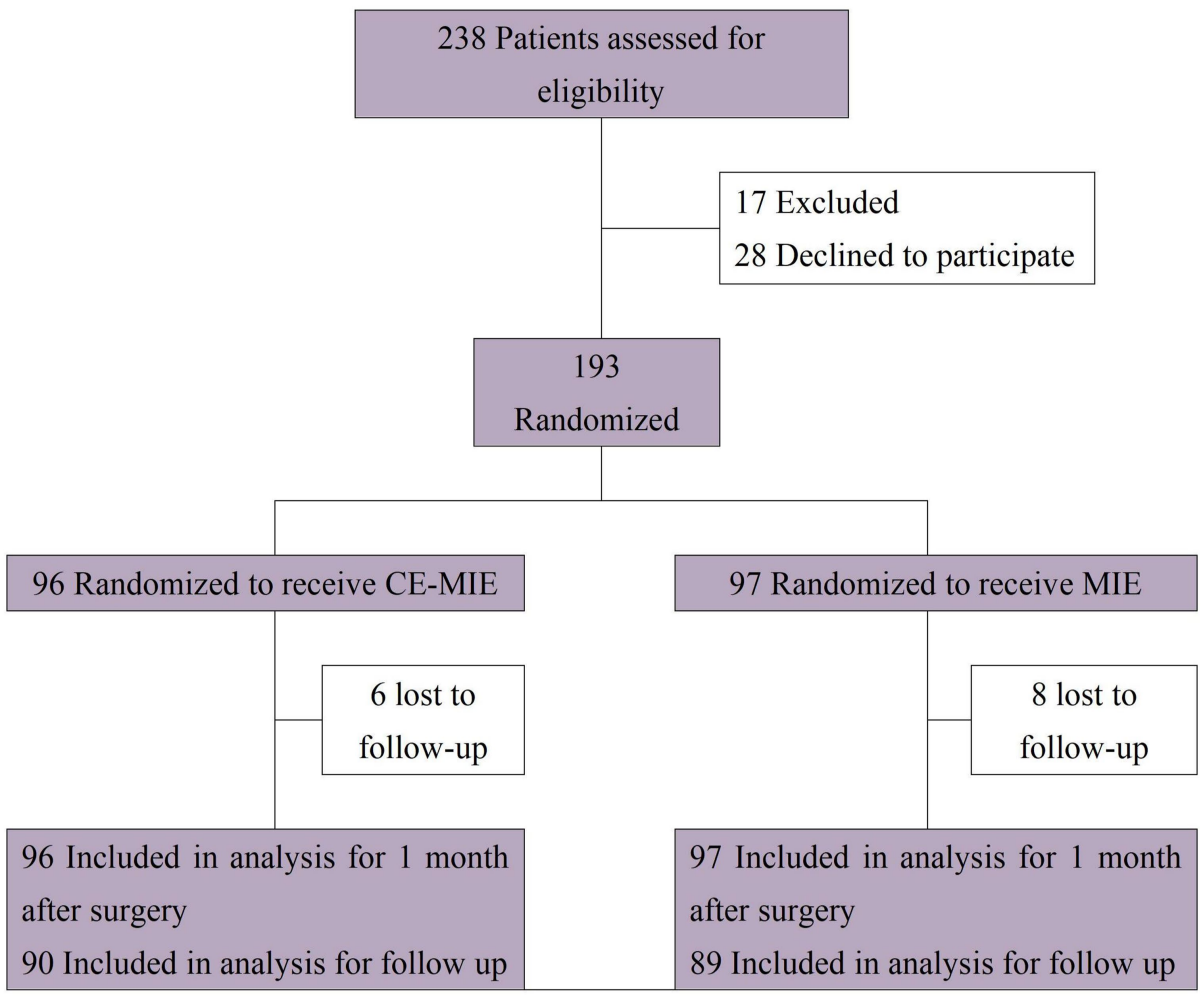
Flow of participants in each group.

Between October 2022 and February 2025, a total of 238 patients with great saphenous varicose veins were recruited at West China Hospital. After excluding 17 patients and 28 patients who declined participation due to inability to read Chinese materials or attend follow-up visits, 193 patients were randomized. Of these, 96 were assigned to the intervention group and 97 to the control group. Six patients (6.25%) in the intervention group and eight patients (8.25%) in the control group were lost to follow-up at 1 year post-surgery. However, data up to 1 month post-surgery were available for all patients (Figure 1).

Females accounted for 53.88% of the participants (56.25% in the intervention group, 51.54% in the control group; *p* = 0.305). The median age was 62 years (range: 57–69) in the intervention group and 66 years (range: 56–72) in the control group, with no statistically significant difference (*p* = 0.240). The median Body Mass Index (BMI) was 24.21 (range: 22.51–26.20) in the intervention group and 24.32 (range: 23.28–27.04) in the control group, showing no significant difference (*p* = 0.319). The proportion of patients in CEAP clinical classes C2-C3 was 67.71% in the intervention group and 61.86% in the control group (*p* = 0.236). Baseline assessments for quality of life and Venous Clinical Severity Score (VCSS) showed no significant differences between the groups (Table 3).

**Table 3:** Baseline characteristics (*n* = 193).

Variables	Intervention group(*n* = 96)	Control group(*n* = 97)	*t*/*X*^2^/*Z*	*p*
Gender			0.430	0.305
Male	42	47		
Female	54	50		
Age	62 (57, 69)	66 (56, 72)	1.175	0.240
BMI(kg/m^2^)	24.21 (22.51, 26.20)	24.32 (23.28, 27.04)	0.997	0.319
CEAP-C			4.709	0.236
2	18	11		
3	47	49		
4	20	26		
5	11	9		
6	0	2		
CIVIQ	61.11 ± 5.31	60.47 ± 5.52	0.821	0.413
VCSS	7 (6.25, 8)	7 (6, 8)	0.716	0.474

BMI, body mass index; CEAP, classification of clinical—etiology—anatomy—pathology; CIVIQ, chronic venous insufficiency questionnaire; VCSS, venous clinical severity score.

### 3.2 Comparison of quality of life

**Figure 2:**
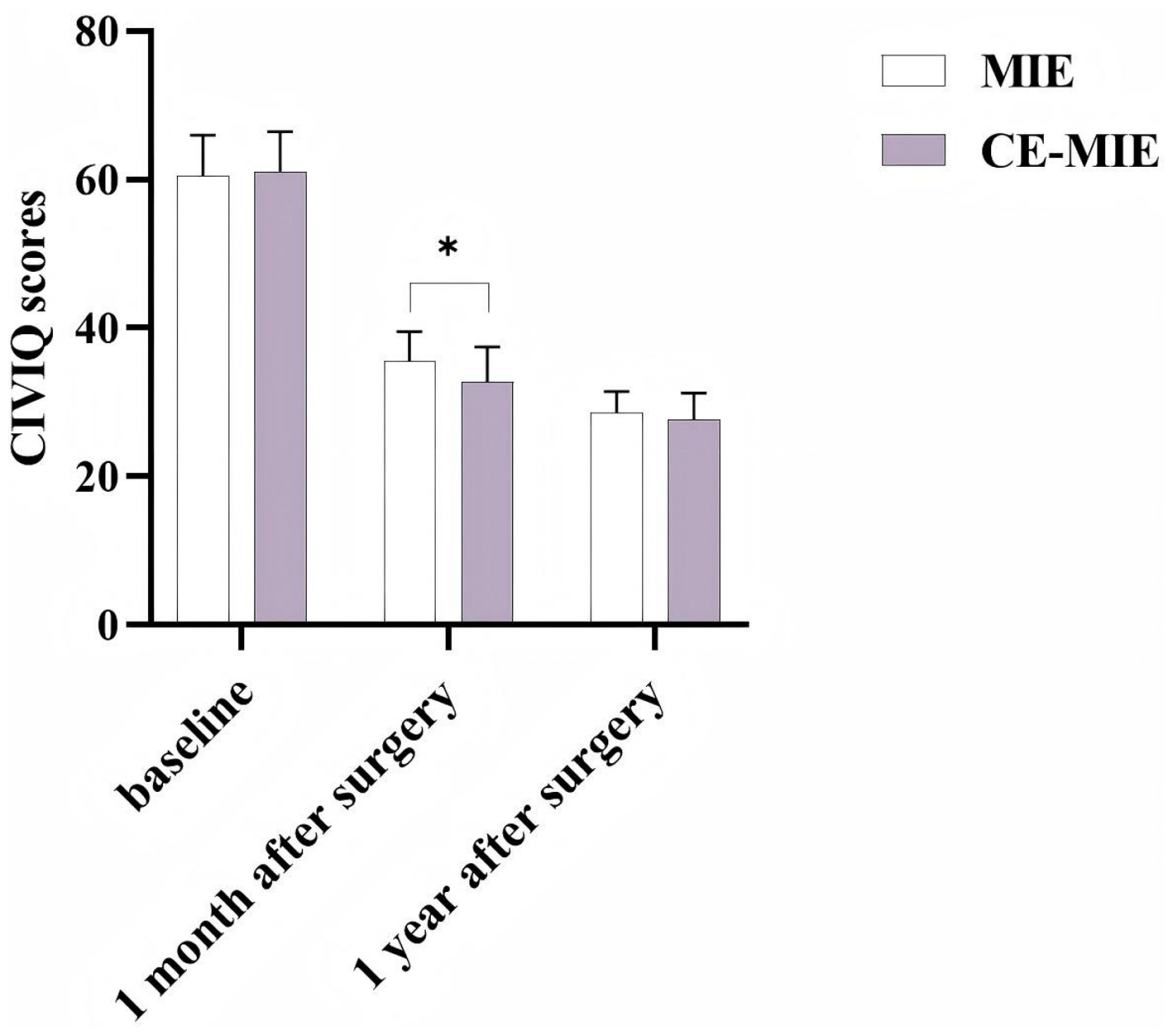
Chronic venous insufficiency questionnaire (CIVIQ) scores results. *p < 0.05.

At 1 month post-surgery, the quality of life score was 32.74 ± 4.72 in the intervention group and 35.49 ± 4.01 in the control group. This difference was statistically significant (*t* = 4.367, *p* = 0.000). At 1 year post-surgery, the quality of life score was 27.71 ± 3.50 in the intervention group and 28.58 ± 2.86 in the control group, a difference that was not statistically significant (*t* = 1.830, *p* = 0.069) (Figure 2).

### 3.3 Comparison of local complications

The incidence of local complications (ecchymosis, redness, and induration) at 3 days, 7 days, and 1 month post-surgery showed no statistically significant differences between the two groups (Table 4).

**Table 4:** Local complications after surgery (*n* = 193).

Variables	Intervention group(*n* = 96)	Control group(*n* = 97)	*X* ^2^	*p*
3 days				
Ecchymosis	51	54	0.126	0.773
Redness	33	33	0.003	1.000
Induration	19	17	0.163	0.715
1 week				
Ecchymosis	28	29	0.012	1.000
Redness	7	13	1.939	0.237
Induration	11	14	0.379	0.669
1 month				
Ecchymosis	10	11	0.042	1.000
Redness	2	8	3.732	0.100
Induration	9	12	0.477	0.645

### 3.4 Comparison of pain score

**Figure 3:**
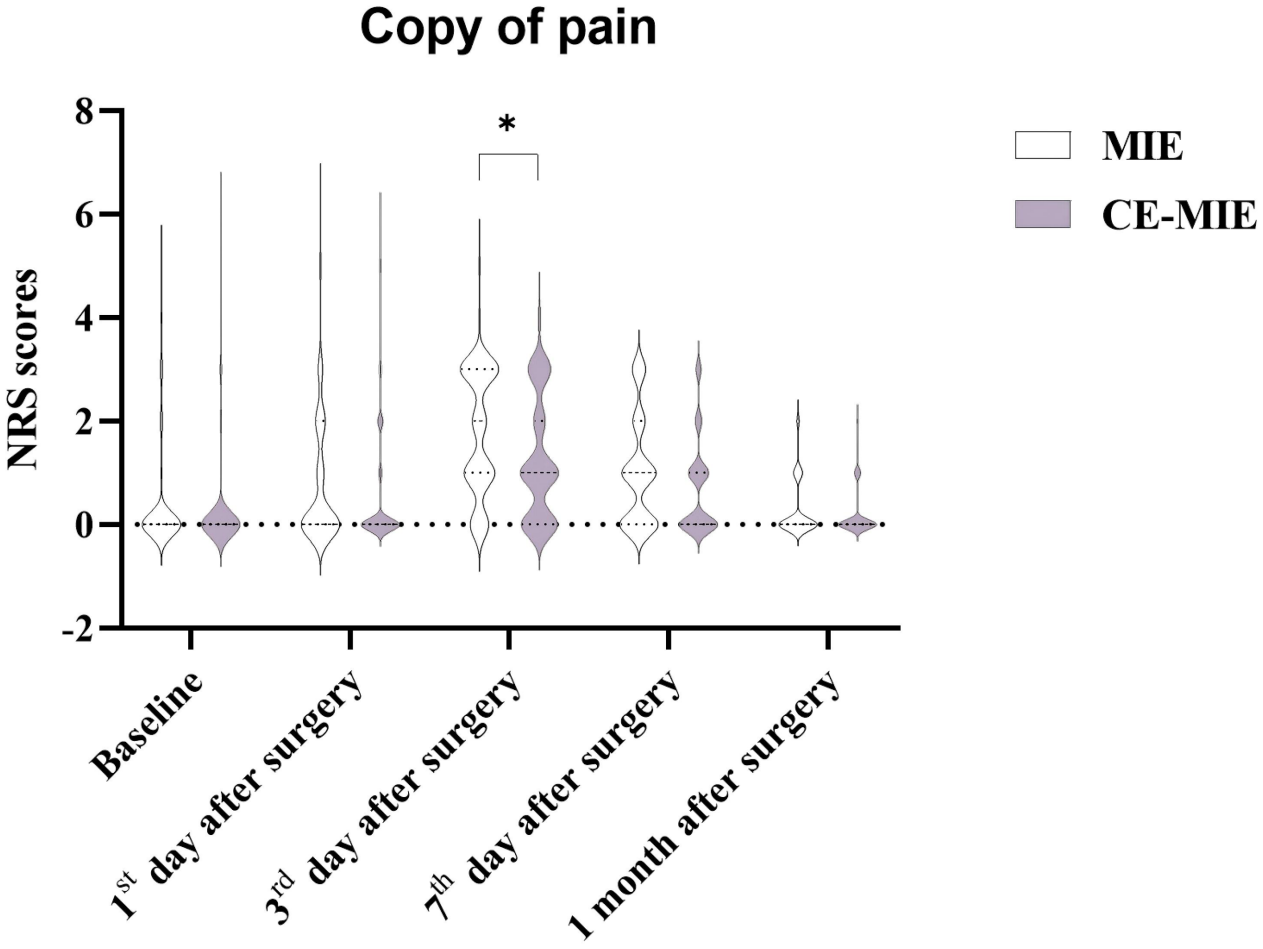
The numeric rating scale (NRS) scores results. *p < 0.05.

No statistically significant difference in pain scores was observed between the two groups at 1 day [Intervention: median 0 (IQR 1); Control: median 0 (IQR 2); *Z* = −1.812, *p* = 0.070] and 1 month post-surgery [Intervention: median 0 (IQR 0); Control: median 0 (IQR 0); *Z* = −1.296, *p* = 0.195]. However, at 3 days post-surgery, the intervention group reported significantly lower pain scores than the control group [Intervention: median 1 (IQR 2); Control: median 2 (IQR 2); *Z* = −2.939, *p* = 0.003]. At 7 days post-surgery, the intervention group had a median pain score of 0 (IQR 1), while the control group had a median score of 1 (IQR 2) (*Z* = −2.199, *p* = 0.028). Although this indicated a favorable trend towards pain reduction, the difference did not meet the adjusted statistical significance threshold (*p* < 0.0125) following the Bonferroni correction (Figure 3).

## 4 Discussion

This study suggests that the CE-MIE model is associated with a significant short-term improvement in QoL compared to the standard MIE model. At 1 month post-surgery, the CE-MIE group showed significantly better QoL outcomes (32.74 ± 4.72 vs. 35.49 ± 4.01, *p* = 0.000). This rapid improvement reflect the multifaceted benefits of the CE-MIE approach across physical, psychological, and social dimensions of patient recovery.

### 4.1 Eliminating uncertainty and reducing anxiety

The use of checklists has been shown to support the standardization and completeness of educational quality (29–31). Checklists also improve communication between patients and healthcare professionals (32), thereby potentially reducing the risk of critical steps are missed. It simplifies the complex rehabilitation process into clear, actionable steps, providing a structured framework for nursing services. This standardization not only ensures operational safety and improves process efficiency, but also significantly boosts patients’ confidence in their recovery. By reducing the cognitive burden on patients during the acute recovery phase, the checklist allows them to focus more on adhering to medical instructions and practicing the necessary skills (33). Overall, this approach appears to contribute to alleviates anxiety and is associated with improved rehabilitation outcomes.

### 4.2 Alleviating pain and physical symptoms

Previous research has highlighted the benefits of elastic compression stockings (ECS) in relieving post-operative edema and venous congestion, thus reducing swelling and discomfort (34). However, a systematic review identified five overarching themes for non-compliance with compression therapy: physical limitations, health literacy, discomfort, financial issues, and psychosocial issues (18). To address these multi-faceted, the dual verification mechanism in this study-combining quantitative skill achievement (score ≥80) with a structured checklist is designed to support that patients not only understand the rationale for ECS but also master precise donning techniques. This may facilitate a transition from passive compliance to informed commitment, motivating them to overcome inherent discomfort. Consequently, this educational approach is associated with reduced limb pain and edema caused by improper wear, potentially alleviating the patients’ physical symptom burden.

### 4.3 Timely feedback and interactive support

The interactive support system, including open-ended Q&A sessions and daily centralized feedback, appeared to play an important role in addressing personalized patient concerns during the acute recovery phase. These sessions were associated with earlier mobilization and supported functional recovery by allowing healthcare providers to respond promptly to emerging issues. Structured tools like Question Prompt Lists (QPLs) have been shown to improve patient engagement by increasing both the frequency and quality of questions (35). This two-way communication reinforces the patient-centered care model and highlights the importance of interactive mechanisms in facilitating better patient outcomes.

### 4.4 Enhancing self-efficacy and sense of control

The dual verification system required patients to actively demonstrate their mastery of key rehabilitation skills, fostering a sense of control over their recovery. This shift from passive recipients of care to active participants empowered patients, enhancing their self-efficacy (36). By encouraging patients to take an active role in their rehabilitation, the CE-MIE model was associated with reduced physical discomfort through skill acquisition. Additionally, it alleviates psychological anxiety by providing clear, structured guidance. Overall, this approach fosters a sense of control, which is crucial for long-term well-being.

### 4.5 One-year outcomes and challenges to sustained quality of life

While the CE-MIE group showed a significant improvement in QoL at 1 month post-surgery, the difference in QoL scores between the two groups at the 1-year follow-up was no longer statistically significant (27.71 ± 3.50 vs. 28.58 ± 2.86, *p* = 0.069). This suggests that while the CE-MIE model provides superior early-stage recovery support, its clinical advantage may diminish as patients’ health statuses naturally converge during 1-year physiological healing process.

### 4.6 Attenuation of intervention effects over 1 year

The checklist’s effectiveness appeared most pronounced during the perioperative period under professional supervision. After discharge, its impact may largely depends on the patient’s ability to manage long-term lifestyle factors such as weight, exercise, and adherence to ECS. Research indicates that the effects of health interventions tend to diminish over time (37, 38). For instance, studies on cardiovascular patients found that while a “Learning and Coping” strategy improved short-term outcomes, it failed to sustain benefits at the 1-year follow-up (10, 39). This suggests that ongoing, theory-based remote support is crucial to mitigate attenuation of behavioral maintenance and enhance 1-year effects. Future health education models should consider integrating continuous post-discharge support to address this challenge (37).

### 4.7 Convergence of baseline effects

At 1 year post-surgery, both groups showed significant improvements in venous disease symptoms, leading to a convergence in their overall health statuses. This phenomenon may be attributed to a “catch-up effect” in the self-care proficiency of the control group. While the CE-MIE group achieved rapid technical mastery early in the study, patients in the MIE group likely reached a comparable level of proficiency through prolonged and repetitive practice. This hypothesis is supported by recent evidence suggesting that postoperative duration significantly influences self-management ability (*F* = 12.534, *p* < 0.001). Patients more than 6 months post-surgery reported significantly higher scores than those at 1–3 months, with a mean difference of 6.77(*p* < 0.001) (40). As patients in both groups naturally improve their management skills over time and as venous symptoms stabilize, the incremental benefits of the enhanced educational model become less apparent.

Consequently, the observed mean difference of 0.87 points at 1 year is likely below the MCID for the CIVIQ-20 instrument. Currently, a consensus on the definitive MCID for the CIVIQ-20 in postoperative populations remains to be established. However, considering the scale’s structure, the observed 0.87-point difference at 1 year accounts for less than 1% of the total score range (20–100) and only a 3.1% relative variance between the group means. This marginal difference suggests that while the CE-MIE group maintained a slight numerical advantage, both interventions yielded clinically comparable long-term outcomes as patients reached a recovery plateau.

### 4.8 Effectiveness of the control group’s basic intervention

The multimedia resources and interactive Q&A platform utilized by the MIE group may have effectively addressed foundational informational needs. By the one-year follow-up, patients in the control group may have developed similar self-care proficiency through repeated practice and gradual adaptation. This process could have contributed to narrowing the initial gap in QoL between the two groups.

### 4.9 Participant attrition and potential bias

At the 1-year follow-up, dropout rates were 6.25% (6 out of 96) in the CE-MIE group and 8.25% (8 out of 97) in the MIE group, indicating a balanced distribution between the two arms (*p* > 0.05). The primary factors contributing to attrition were loss of contact, which appeared to be independent of the intervention itself. To assess potential bias, we compared the baseline characteristics of the dropouts to those who completed the study. No significant differences were identified, suggesting that the attrition was likely missing at random. Nonetheless, we recognize that the slight narrowing of the QoL gap at the 1-year mark may be partially influenced by the loss of these participants. We recommend that future studies incorporate more robust retention strategies to further mitigate the impact of attrition bias.

### 4.10 Pain and objective complications assessment

Significantly lower pain scores were sustained at 3 days post-surgery, even after applying the Bonferroni adjustment. By day 7, although the statistical difference had diminished (*p* = 0.028) following the Bonferroni correction, the intervention group still exhibited a clinical trend toward lower median pain scores. Initial post-operative pain is typically influenced by surgical trauma, residual anesthetic effects, and intraoperative analgesia, making it less likely that the educational intervention would have a marked effect on Day 1. Research indicates that post-operative pain peaks on Day 1 and rapidly declines thereafter (41, 42). The real efficacy of the CE-MIE model is seen during the rehabilitation and self-management phase. The observed superior pain relief in the CE-MIE group at 3 days post-surgery is likely associated with the checklist’s enforcement of skill mastery through dual verification, transforming patients’ knowledge into correct execution. This mechanism may have helped minimize painful complications associated with improper self-management techniques. Proper pain management in the immediate post-surgery period is crucial, as studies have shown that severe acute pain is a predictor of chronic post-operative pain (43). By alleviating short-term pain, CE-MIE may help reduce the long-term risk of chronic pain.

There were no statistically significant differences in the incidence of local complications between the two groups at 3 days, 7 days, or 1 month post-surgery. This suggests that both groups, through their shared requirement of achieving a minimum score for ECS wear (≥80 points), exhibited similar effectiveness in managing objective complications (34). However, the CE-MIE model appeared to show more benefit in subjective measures, such as pain and QoL, than in objective complication rates.

Regarding the study’s design, the observed variance of the CIVIQ scores was notably lower than the initial assumptions used for sample size estimation. The pooled standard deviation at the primary follow-up point (1 month) was 4.38, in contrast to the assumed value of 8.41. This reduction in variance allowed the study to maintain high statistical power, despite a mean difference that was slightly smaller than anticipated. Consequently, this reinforces the reliability of our findings.

### 4.11 Limitations of the study

This study was conducted at a single center, which may limit the generalizability of its findings. The core focus of the intervention (checklist enhancement) centered on standardizing perioperative processes and ensuring skill achievement, but it lacked a designed mechanism to maintain behavior change after discharge. This limitation likely contributed to the inability to sustain the QoL advantage in the long term. Furthermore, while we monitored pain levels during the acute recovery phase, the study design did not include an assessment of pain at the 1-year follow-up. This prevents us from evaluating whether the educational intervention has a lasting impact on chronic pain management or potential late-onset discomfort. Furthermore, this study primarily relied on Patient-Reported Outcome Measures (PROMs), such as pain and QoL, which may be influenced by various factors, including psychological status, patient expectations, and recall bias. Although we monitored objective clinical indicators, such as local complication rates, to ensure safety, the absence of physiological data represents a limitation. Future research should incorporate these objective physiological measures to offer a more comprehensive evaluation of the intervention’s efficacy.

### 4.12 Future research directions

To maintain long-term benefits observed in the early postoperative stages, future research should aim to extend structured intervention models into the post-discharge recovery phase. Additionally, the scope should be broadened to encompass a wider population of patients with CVD. While this study focused on perioperative recovery, future programs should prioritize early prevention of complications, such as venous ulcers and deep vein thrombosis, across all stages of CVD.

Our one-year results revealed a convergence in quality of life scores between groups, suggesting a need for ongoing support to mitigate intervention decay. Digital health solutions could effectively bridge this gap. AI-driven hybrid chatbots have the potential to enhance patient engagement and service delivery (44). These interactive conversational agents offer real-time, personalized planning and advice, which can help improve patient behaviors (45). By integrating technology-supported remote guidance with the standardized in-hospital CE-MIE model, future programs may foster long-term adherence to self-care practices and complication monitoring. This shift toward a comprehensive, multi-stage health education framework would benefit not only operative recovery but also the preventive management of the wider population affected by chronic venous disease.

## 5 Conclusion

Checklist-enhanced multimedia interactive education (CE-MIE) significantly improves acute post-operative pain management and notably enhances quality of life scores at 1 month after surgery in patients with lower extremity varicose veins. It is recommended that structured checklists be integrated into clinical nursing practice to standardize perioperative health education, ensuring that patients acquire essential self-care skills and achieve optimal early rehabilitation. This approach also lays the groundwork for developing future health education models that include continuous support after discharge.

## Data Availability

The raw data supporting the conclusions of this article will be made available by the authors, without undue reservation.
